# Antibiotic and heavy-metal resistance of *Vibrio parahaemolyticus* isolated from fresh shrimps in Shanghai fish markets, China

**DOI:** 10.1007/s11356-016-6614-4

**Published:** 2016-04-16

**Authors:** Yu He, Lanlan Jin, Fengjiao Sun, Qiongxia Hu, Lanming Chen

**Affiliations:** Key Laboratory of Quality and Safety Risk Assessment for Aquatic Products on Storage and Preservation (Shanghai), China Ministry of Agriculture, College of Food Science and Technology, Shanghai Ocean University, 999 Hu Cheng Huan Road, Shanghai, 201306 Peoples’ Republic of China

**Keywords:** *Vibrio parahaemolyticus*, Aquatic products, Virulence, Antimicrobial resistance, Heavy metal resistance, Pulsed-field gel electrophoresis

## Abstract

*Vibrio parahaemolyticus* is a causative agent of human serious seafood-borne gastroenteritis disease and even death. Shrimps, often eaten raw or undercooked, are an important reservoir of the bacterium. In this study, we isolated and characterized a total of 400 *V. parahaemolyticus* strains from commonly consumed fresh shrimps (*Litopenaeus vannamei*, *Macrobrachium rosenbergii*, *Penaeus monodon*, and *Exopalaemon carinicauda*) in Shanghai fish markets, China in 2013–2014. The results revealed an extremely low occurrence of pathogenic *V. parahaemolyticus* carrying two major toxic genes (*tdh* and *trh*, 0.0 and 0.5 %). However, high incidences of antibiotic resistance were observed among the strains against ampicillin (99 %), streptomycin (45.25 %), rifampicin (38.25 %), and spectinomycin (25.50 %). Approximately 24 % of the strains derived from the *P. monodon* sample displayed multidrug resistant (MDR) phenotypes, followed by 19, 12, and 6 % from the *E. carinicauda*, *L. vannamei*, and *M. rosenbergii* samples, respectively. Moreover, tolerance to heavy metals of Cr^3+^ and Zn^2+^ was observed in 90 antibiotic resistant strains, the majority of which also displayed resistance to Cu^2+^ (93.3 %), Pb^2+^ (87.8 %), and Cd^2+^(73.3 %). The pulsed-field gel electrophoresis (PFGE)-based genotyping of these strains revealed a total of 71 distinct pulsotypes, demonstrating a large degree of genomic variation among the isolates. The wide distribution of MDR and heavy-metal resistance isolates in the PFGE clusters suggested the co-existence of a number of resistant determinants in *V. parahaemolyticus* population in the detected samples. This study provided data in support of aquatic animal health management and food safety risk assessment in aquaculture industry.

## Introduction

China has become the world’s largest producer of aquatic products since 2002 (People’s Republic of China, Fishery Products Annual Report). Along with the fast growing aquaculture industry, however, aquatic animal diseases have also rapidly increased (Wu et al. 2014). Antimicrobial agents are commonly used in the animal breeding industry and effectively prevent disease outbreaks caused by pathogenic microorganism. Nevertheless, the inappropriate usage of antimicrobial drugs in aquaculture contributed to the development of antibiotic-resistant bacteria and imposed serious problems on aquatic ecosystems, particularly in the developing countries (Woolhouse and Farrar 2014). For example, the high incidences of resistance to antimicrobial agents such as ampicillin, rifampicin, and streptomycin have been reported in *V. parahaemolyticus* isolates originated from some aquatic products in Asian and European countries, e.g., southern China (Xie et al. 2015), Korea (Kang et al. 2015), Poland (Lopatek et al. 2015), and Italy (Ottaviani et al. 2013). On the other hand, in addition to increasing industrialization, environmental pollution has become one of the most challenging issues in the developing countries. High occurrence of heavy metal resistant bacteria has been detected in various environments, e.g., marine, river and agricultural soil (Sabry et al. 1997; Ansari et al. 2008; Malik and Aleem 2011). Contaminated water with industrial pollutants (e.g., heavy metals) was supposed to enhance the selection for antibiotic resistance and vice versa (An et al. 2010; Matyar 2012; Zhao et al. 2012).

*V. parahaemolyticus* is a Gram-negative, halophilic bacterium that thrives in marine, estuarine, and aquaculture environments worldwide (Broberg et al. 2011; Letchumanan et al. 2014). The bacterium is a causative agent of serious human seafood-borne gastroenteritis disease and even death (Boyd et al. 2008; Ceccarelli et al. 2013). In China, the incidence of food-borne illnesses caused by consumption of aquatic products contaminated with *V. parahaemolyticus* has become one of the most important food safety risk, particularly in the southeast littoral provinces (Wang et al. 2007; Chen et al. 2010). Shrimps, often eaten raw or undercooked, are an important reservoir of *V. parahaemolyticus*. To date, numerous studies have been conducted to characterize *V. parahaemolyticus* from clinical samples in different parts of the world (e.g., Boyd et al. 2008; Broberg et al. 2011; Ceccarelli et al. 2013; Tsai et al. 2013; Letchumanan et al. 2014); nevertheless, insufficient information is available on the isolates from aquaculture products, such as various shrimps in China (e.g., Chen et al. 2012; Song et al. 2013; Xu et al. 2014; Albuquerque Costa et al. 2015; Xie et al. 2015). Thus, in this study, we aimed to determine antibiotic and heavy-metal resistance of the 400 *V. parahaemolyticus* strains isolated from four types of fresh shrimps commonly consumed in Shanghai, China, in order to address the lack of molecular ecological data of the bacterium in aquaculture products.

## Materials and methods

### Sample collection

The fresh shrimps, including *L. vannamei, M. rosenbergii*, *P. monodon*, and *E. carinicauda*, were collected monthly from Shanghai fish markets in Shanghai, China from June to November in 2013 and 2014. The former three are widely cultured in the southeast littoral provinces in China, while the latter is a type of small shrimp grown in Shanghai and neighboring areas. The *L. vannamei* (known as Pacific white shrimp) is the most widely cultured and productive alien crustacean worldwide. It is native to the western Pacific coast of Latin America and introduced commercially since 1996 into China and several countries in Asia. The freshwater culture of *L. vannamei* has proven even more successful than brackish water culture conditions (Tang et al. 2014). *M. rosenbergii* (known as the giant river prawn) is the most important cultured freshwater prawn in the world. It is native to the Indo-Pacific region, northern Australia, and Southeast Asia, and now farmed on a large scale in many countries (Sahul Hameed and Bonami 2012). *P. monodon* (known as the black tiger shrimp) is a marine crustacean especially widely cultured in its natural distribution region of Indo-Pacific (Nunan et al. 2005). *E. carinicauda* is widely distributed in the East China Sea. It is one of the major economic shrimp species cultured in China (Zhang et al. 2015). The samples stored in sterile plastic bags (Shanghai Sangon Biological Engineering Technology and Services Co., Ltd., Shanghai, China) were immediately transported in icebox to our laboratory at Shanghai Ocean University in Shanghai, China for experiments.

### Isolation and identification of *V. parahaemolyticus* isolates

*V. parahaemolyticus* was isolated and identified according to the instructions of the Chinese Government Standard (GB17378-2007) and the Standard of the Bacteriological Analytical Manual of the US Food and Drug Administration (8th Edition, Revision A, 1998) (Song et al. 2013). Briefly, aliquots (25 g) of each shrimp sample were individually homogenized in appropriate volumes of alkaline peptone water (APW, Beijing Land Bridge Technology Co., Ltd., Beijing, China) using the lab blender BagMixer (Interscience, Paris, France). Microbial cells in supernatant were appropriately diluted and spread on the CHROMagar^TM^ Vibrio (CHROMagar, Paris, France) or thiosulfate citrate bile salts sucrose (TCBS, Beijing Land Bridge Technology Co., Ltd., Beijing, China) agar plates. The plates were incubated at 37 °C for 24 h. Colonies were picked out, screened, and identified according to the method described previously (Song et al. 2013). Genomic DNA preparation, oligonucleotide primer synthesis, PCR reactions and sequence analysis were performed as previously described (Song et al. 2013; Tang et al. 2014). The virulence genes (*tdh* and *trh*) were detected by PCR as previously described (Song et al. 2013). *V. parahaemolyticus* ATCC33847 (*tdh*^+^*trh*^−^) (Fujino et al. 1965) and ATCC17802 (*tdh*^−^*trh*^+^) (Baumann et al. 1973), isolated from clinical and food-poisoning cases, respectively, were used as positive control strains as described previously (He et al. 2015).

### Susceptibility to antimicrobial agents and heavy metals

*V. parahaemolyticus* isolates were measured for in vitro susceptibility to ten antimicrobial agents using Kirby-Bauer disk diffusion method according to the Clinical and Laboratory Standards Institute (CLSI, 2006, Approved Standard-Ninth Edition, M2-A9, Vol. 26 No. 1) (Song et al. 2013). Mueller-Hinton agar medium (Oxoid, UK) and the disks with antimicrobial agents (Oxoid, UK) were used in this study, including 10-μg ampicillin (AMP), 30-μg chloramphenicol (CHL), 10-μg streptomycin (STR), 10-μg gentamicin (CN), 30-μg kanamycin (KAN), 5-μg rifampicin (RIF), 100-μg spectinomycin (SPT), 30-μg tetracycline (TET), 5-μg trimethoprim (TM), and 25-μg SXT (sulfamethoxazole (23.75 μg)-trimethoprim (1.25 μg)). Susceptible, intermediate, and or resistant phenotypes were reported according to the established breakpoints for *V. parahaemolyticus*. In the case of the lacking of the established breakpoints of some antimicrobial agents for the bacterium, the values for *Vibrio cholerae* or enterobacteriaceae were referred. To date, no standard method is available to measure bacterial susceptibility to heavy metals. Tolerance of the isolates to heavy metals was determined according to the method described previously (Malik and Aleem 2011; Song et al. 2013). The minimal inhibitory concentration (MIC) in vitro of the tested heavy metals against the isolates was measured quantitatively using Broth Dilution Testing (microdilution) (CLSI, 2006). The heavy metals used in this study included NiCl_2_, CrCl_3_, CdCl_2_, PbCl_2_, CuCl_2_, ZnCl_2_, MnCl_2_, and HgCl_2_ [Analytical Reagent (AR), Sinopharm Chemical Reagent Co., Ltd, Shanghai, China]. The assays were performed in triplicate experiments, and quality control strains of *Escherichia coli* ATCC25922 and K12 were purchased from the Institute of Industrial Microbiology (Shanghai, China), and used in the antibiotic and heavy-metal resistance tests, respectively (Malik and Aleem 2011; Song et al. 2013).

### PFGE-based genotyping analysis

The PFGE analysis was performed according to the method described previously (He et al. 2015). Genomic DNA fragments digested with the restriction endonuclease *Not*I (Japan TaKaRa BIO, Dalian Company, Dalian, China) were resolved in a CHEF Mapper system (Bio-Rad Laboratories, Hercules, Calif., USA). Chromosome DNA of *Salmonella enterica* strain H9812 was digested with the restriction endonuclease *Xba*I (Japan TaKaRa BIO, Dalian Company, Dalian, China) and used as DNA molecular markers ranging from 20.5 to 1,135 kb. PFGE patterns were analyzed using the NTSYSpc 2.10e Software according to the unweighted pair group method with arithmetic mean based on Dice coefficients.

## Results and discussion

### Virulence of the *V. parahaemolyticus* isolates

*L. vannamei, M. rosenbergii*, *P. monodon*, and *E. carinicauda* are very common shrimps consumed in Shanghai, China. Pure culture of randomly selected 100 *V. parahaemolyticus* strains isolated from each type of the shrimps was analyzed in this study. Pathogenic *V. parahaemolyticus* produces two major toxic proteins, thermostable direct haemolysin (TDH) and TDH-related haemolysin (TRH), which play a crucial role in the diarrhea disease elicited by the bacterium (Boyd et al. 2008). In this study, a total of 400 *V. parahaemolyticus* strains were subjected to the detection of the two virulence-associated genes by PCR. The results revealed that all the isolates were featured with no toxic *tdh* gene. However, the *trh* gene was detected positive from two isolates derived from *L. vannamei* and *P. monodon*, respectively. The very low occurrence of pathogenic *V. parahaemolyticus* has also been reported from the majority of non-clinical samples previously (e.g., Chao et al. 2009; Song et al. 2013; Haley et al. 2014).

### Susceptibility of the *V. parahaemolyticus* isolates to antimicrobial agents

**Fig. 1:**
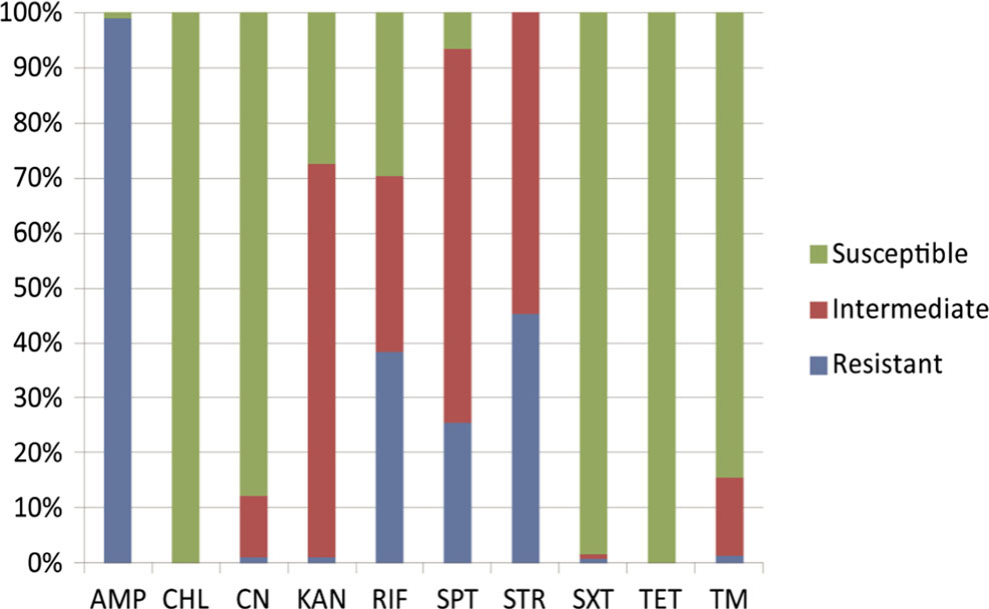
Antimicrobial susceptibility of the four hundred *V. parahaemolyticus* strains isolated from the fresh shrimp samples collected in Shanghai fish markets in 2013–2014. *AMP* ampicillin, *CHL* chloramphenicol, *CN* gentamicin, *KAN* kanamycin, *RIF* rifampicin, *SPT* spectinomycin, *STR* streptomycin, *SXT* sulfamethoxazole-trimethoprim, *TET* tetracycline, *TM* trimethoprim

Antimicrobial susceptibility of the 400 *V. parahaemolyticus* isolates was determined, and ten antimicrobial agents were tested. As illustrated in Fig. 1, all the isolates were susceptible to CHL and TET. Of these, a total of 35 strains showed non-resistance to all the ten drugs. Since 2002, CHL, its salts and esters (including cholramphenicol succinate) have been banned to use in breeding industry in China (China Department of Agriculture, Bulletin No. 193), which may serve as an explanation of the result in this study. Our observation correlated with a recent report (Xie et al. 2015), showing that all 150 *V. parahaemolyticus* isolates in aquatic products collected from South China markets were also susceptible to CHL. Albeit the wide usage of TET, sulfonamides and quinolones in aquaculture has been reported (Holmström et al. 2003), the resistance to TET was not detected from all the *V. parahaemolyticus* isolates in this study. In contrast, consistent with the previous studies (Matyar 2012; Song et al. 2013; Kang et al. 2015), AMP resistance was the most predominant (99 %) among the isolates examined in this study. Moreover, the resistance of the isolates to the other antimicrobial agents was also observed, including STR (45.3 %), RIF (38.3 %), and SPT (25.5 %). Meanwhile, the isolates showed high levels of intermediate susceptibilities to these three drugs (Fig. 1). High incidences of resistance to STR (88.7, 50.7 %) have recently been reported in *V. parahaemolyticus* isolates originating from aquatic products in China (Xie et al. 2015) and Korea (Kang et al. 2015) as well. The STR-resistant *V. parahaemolyticus* isolates in Korea also showed RIF resistant phenotype (50.7 %). As a broad-spectrum antibiotic, SPT is often used in livestock and poultry breeding industry. In this study, about 25.5 % of the isolates exhibited strong resistance phenotype against SPT, which was not detected previously. In addition, very few isolates exhibited tolerance to TM (1.25 %), KAN (1.00 %), CN (1.00 %), and SXT (0.75 %). Nevertheless, a high percentage of intermediate susceptibility to KAN was detected in the isolates (70 %), suggesting a potential resistance trend of this drug.

**Fig. 2:**
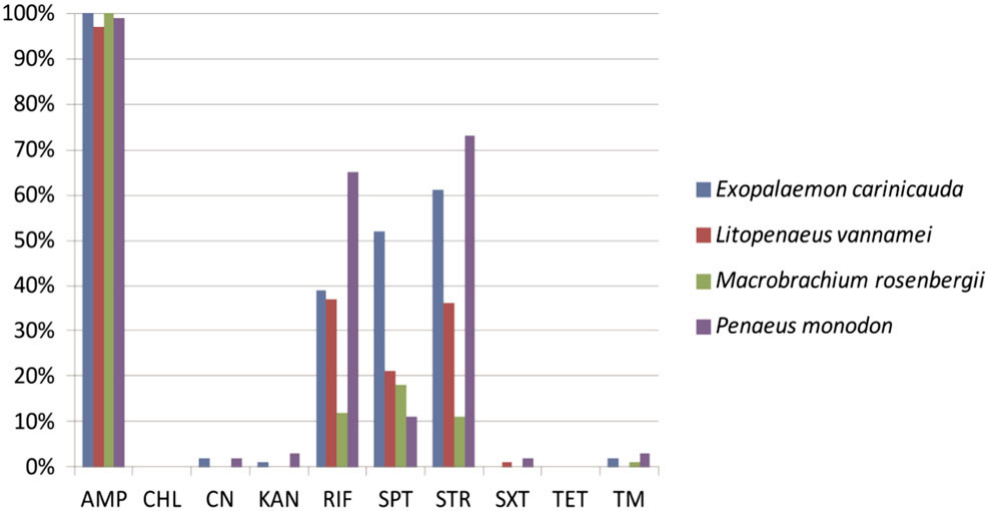
Incidences of antibiotic resistant *V. parahaemolyticus* strains derived from the four different shrimp samples. *AMP* ampicillin, *CHL* chloramphenicol, *CN* gentamicin, *KAN* kanamycin, *RIF* rifampicin, *SPT* spectinomycin, *STR* streptomycin, *SXT* sulfamethoxazole-trimethoprim, *TET* tetracycline, *TM* trimethoprim

As shown in Fig. 2, our results also revealed distinct resistance patterns yielded by the *V. parahaemolyticus* isolates of different shrimp origins. AMP resistance was the most predominant among all the samples (97–100 %). High percentages of AMP resistance have also been observed in the bacterium isolated from *P. monodon* in India (Bhattacharya et al. 2000) and *L. vannamei* in Brazil (Rodrigues de Melo et al. 2011). In this study, the isolates derived from the *P. monodon* sample had the highest resistance levels against STR (73 %) and RIF (65 %) and also exhibited resistance to the maximum number of antimicrobial agents (8/10), whereas those from *M. rosenbergii* showed an opposite pattern. In addition, the highest percentage of SPT resistance was detected from the isolates of *E. carinicauda* origin (52 %), which was notably higher than those from the other three samples (11–21 %). Moreover, the resistance to STR and RIF was the second abundant in the *E. carinicauda* strains, when compared to the other samples. To our knowledge, the comparative antibiotic resistance patterns of *V. parahaemolyticus* isolates have not been described in the four species of shrimps thus far. Moreover, this study constituted the first investigation of *V. parahaemolyticus* strains originated from *M. rosenbergii* and *E. carinicauda*.

**Fig. 3:**
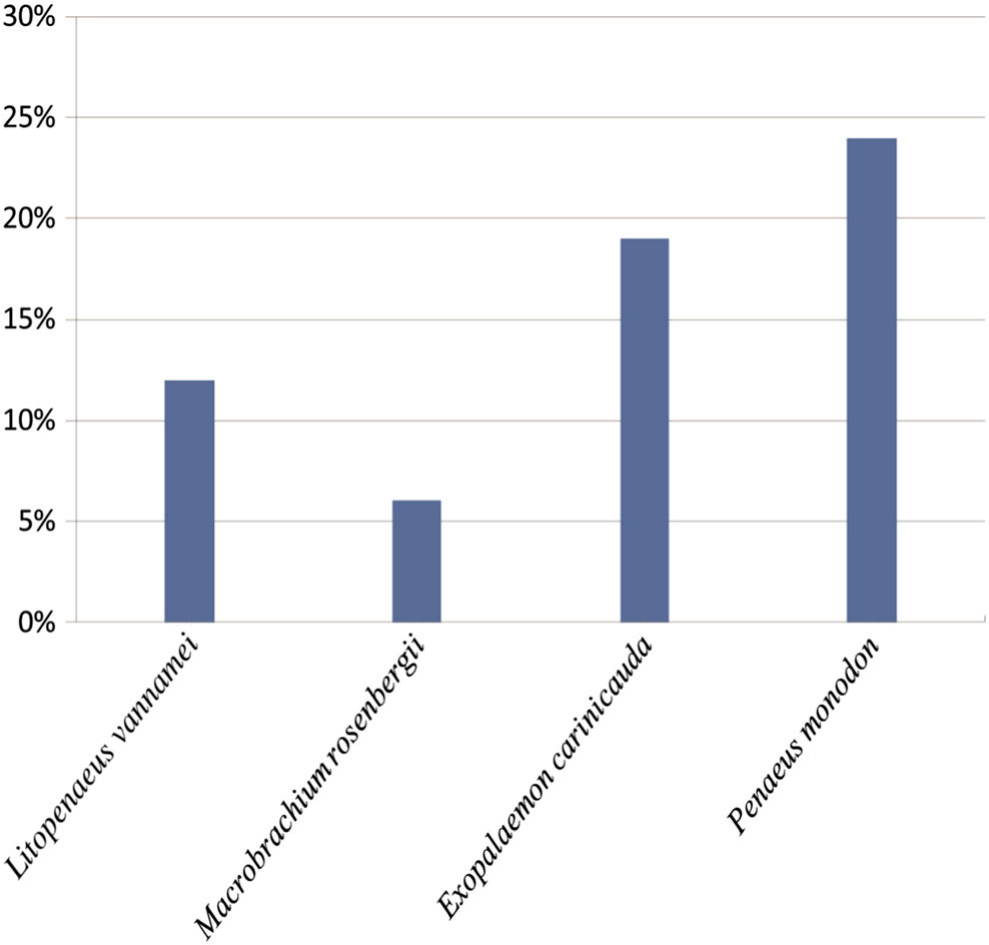
Incidences of the MDR *V. parahaemolyticus* strains in the four different shrimp samples

Multidrug resistance (MDR) was defined as non-susceptibility to at least one agent in three or more antimicrobial categories (Thapa Shrestha et al. 2015). MDR phenotypes have been observed in *Vibrios* derived from *L. vannamei* and *P. monodon* (de Melo et al. 2011; Albuquerque Costa et al. 2015). In this study, approximately 15.3 % of the tested isolates exhibited MDR phenotypes, which varied depending on the shrimp samples. The strains derived from the *P. monodon* sample showed the highest occurrence of MDR (24 %), followed by 19, 12, and 6 % from the *E. carinicauda*, *L. vannamei*, and *M. rosenbergii* samples, respectively (Fig. 3). Taken together, our data revealed the most prevalent antibiotic resistance among the *V. parahaemolyticus* isolates originating from *P. monodon*, which could be a result of serious contamination in this sample source. *P. monodon* is cultured in brackish water conditions, it will be interesting to trace back and investigate the possible reasons for the high prevalence of antibiotic resistance in future research.

### Tolerance of the *V. parahaemolyticus* isolates to heavy metals

**Fig. 4:**
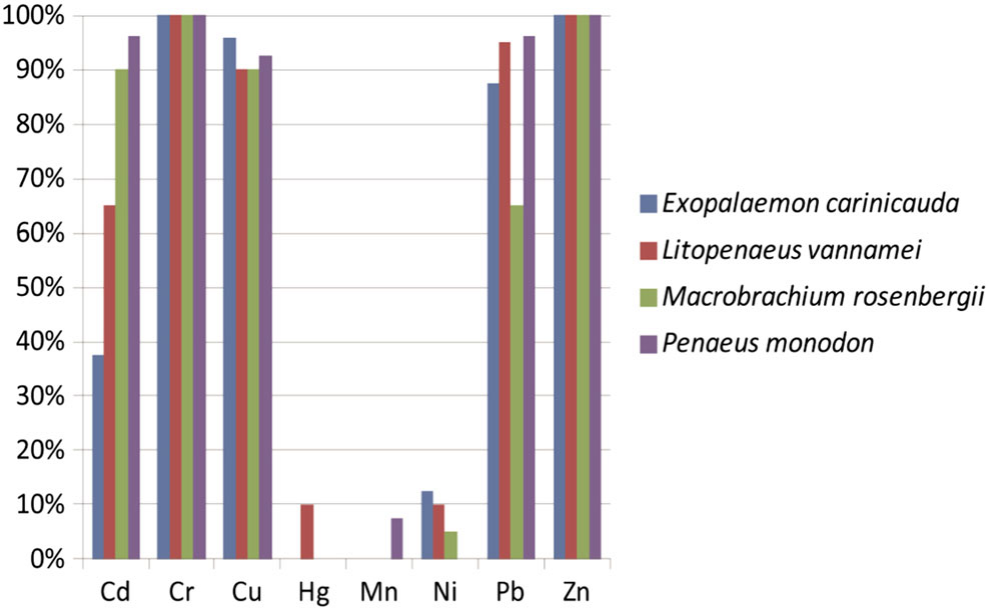
Incidences of heavy metal resistant *V. parahaemolyticus* strains derived from the four different shrimp samples

In this study, based on the antibiotic resistant results, a total of 90 selected antibiotic resistant *V. parahaemolyticus* isolates of the shrimp origin were further examined for their susceptibilities to heavy metals, including Cd^2+^, Cr^3+^, Cu^2+^, Hg^2+^, Mn^2+^, Ni^2+^, Pb^2+^, and Zn^2+^. As shown in Table 1, a maximum MIC of 3200 μg/mL for Cd^2+^, Cr^3+^, Cu^2+^, Mn^2+^, Ni^2+^, Pb^2+^, and 800 μg/ml for Zn^2+^ and 50 μg/ml for Hg^2+^ were observed, when compared to the quality control strain *E. coli* K12 (Malik and Aleem 2011). All the *V. parahaemolyticus* isolates were resistant to Cr^3+^ and Zn^2+^, the majority of which also displayed resistance to Cu^2+^ (93.3 %), Pb^2+^ (87.8 %), and Cd^2+^(73.3 %). In addition, about 6.7 % of the isolates showed resistance to Ni^2+^. It has been reported that the Yangtze River Estuary area has suffered heavy metal contamination, being located in one of the highest density of population and fastest economic developing areas in China (An et al. 2010; Zhao et al. 2012). Heavy-metal resistance has also been observed in *Vibrios* isolated from aquatic products and environment in the Yangze River estuary in Shanghai (Song et al. 2013). In this study, almost all the isolates were susceptible to Mn^2+^ and Hg^2+^, except those isolated from *P. monodon* and *L. vannamei* samples, where very low percentage of the isolates (2.2 %) was detected resistant to the two heavy metals (Fig. 4).

**Table 1:** Incidence of heavy metal resistance among the ninety *V. parahaemolyticus* isolates

Heavy metal	MIC (μg/mL)										Resistant	
	6.25	12.5	25	50	100	200	400	800	1600	3200	*n*	(%)
Cd							^a^					
							24	63	3		66	73.3
Cr								^a^				
									49	41	90	100
Cu								^a^				
								6	33	51	84	93.3
Hg		^a^										
	47	42	1	1							2	2.2
Mn									^a^			
							56	29	3	2	2	2.2
Ni								^a^				
						2	1	81	5	1	6	6.7
Pb									^a^			
									11	79	79	87.8
Zn							^a^					
								90			90	100

^a^Minimal inhibition concentration of standard strain *E. coli* K12

As shown in Fig. 4, the *V. parahaemolyticus* isolates derived from different shrimp sources had similar heavy-metal resistance patterns, most of which displayed resistance to Cr^3+^, Cu^2+^, and Zn^2+^ (90–100 %). Moreover, about 65.0–96.3 % of the isolates showed resistance to Pb^2+^ and Cd^2+^, except the lower percentage of Cd^2+^ resistance in *E. carinicauda* (37.5 %). The results indicated that the sample sources appeared not greatly impact on the major heavy metal resistant patterns of *V. parahaemolyticus*. One possibility was that inappropriate release of industrial wastes may influence on different aquaculture environments.

In this study, our data indicated that the tolerance to heavy metals was very prevalent in the *V. parahaemolyticus* strains with more than two antibiotic resistance phenotypes. Industrial pollutants were supposed to enhance the selection for antibiotic resistance and vice versa (Bhattacharya et al. 2000; Baker-Austin et al. 2006; Malik and Aleem 2011). The abundant double-resistant bacteria could be a cause of serious concern due to the potential health impacts of consuming contaminated products (Holmström et al. 2003; Sharma et al. 2007).

### Phylogenetic relationships of the resistant *V. parahaemolyticus* isolates

**Fig. 5:**
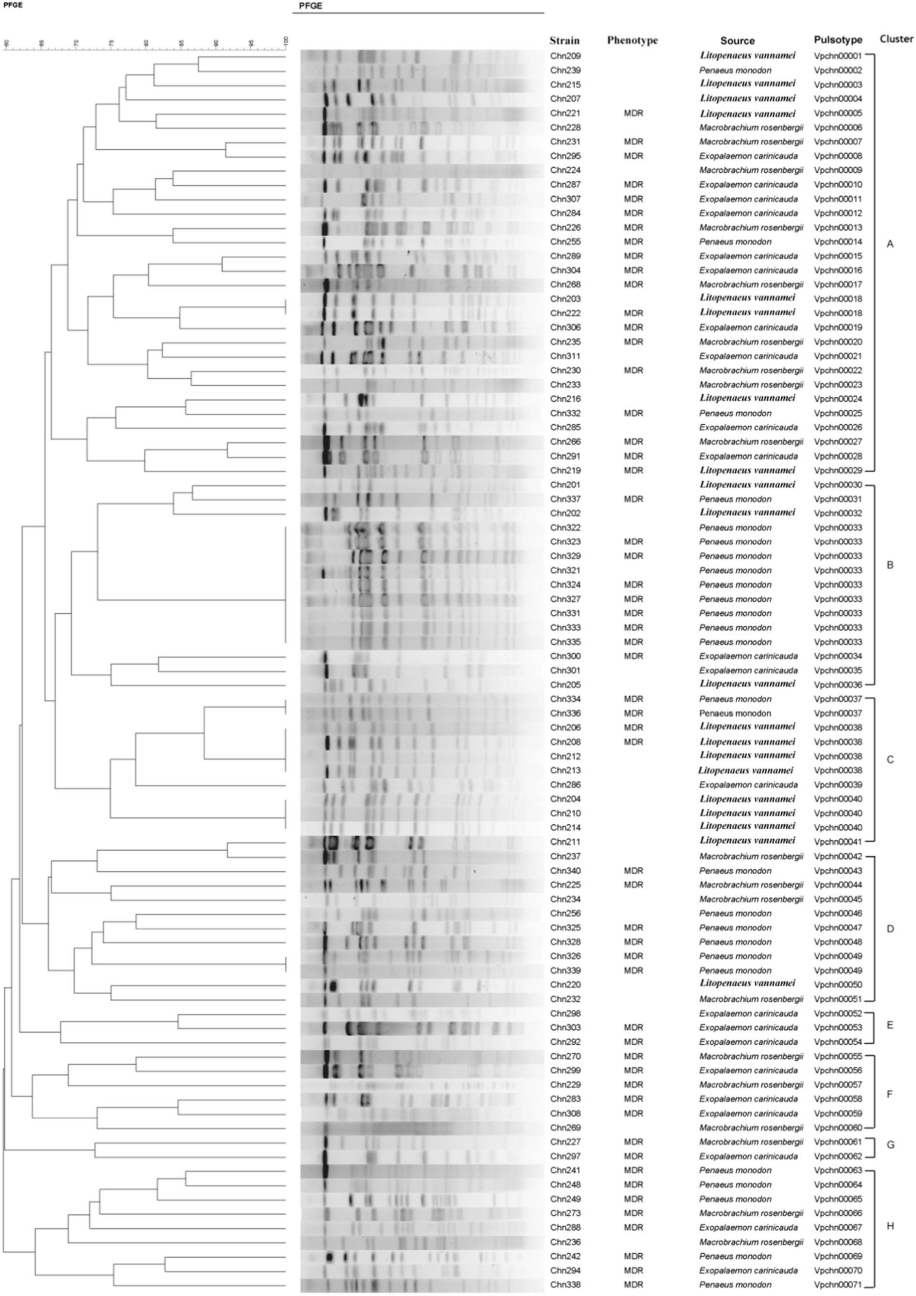
The PFGE-base genotyping of the *V. parahaemolyticus* strains in this study

To track the relatedness of the 90 resistant isolates, we obtained their genome fingerprinting profiles (Fig. 5). Only three isolates could not be examined by the *Not*I-PFGE analysis in this study. Given the significant difference of a single DNA band in size ranging from 20.5 to 1135 kb on the PFGE gels, cluster analysis of the *Not*I-PFGE profiles revealed a total of 71 pulsotypes. Five pairs of isolates and one group of six isolates clustered at ≥87 % similarity, which is a cut-off value that has been suggested for use in identifying isolates belonging to the same epidemic strain (Seifert et al. 2005). The majority of the isolates (81.6 %) shared 60–87 % similarity in this study. In addition, all the isolates were assigned into eight distinct clusters, among which the majority of the isolates (89.7 %) into Cluster A to G, whereas nine isolates into Cluster H, which was more distantly related with the formers (Fig. 5). These results demonstrated that the *V. parahaemolyticus* isolates varied considerably, with remarkable genetic diversity existing in the tested shrimp samples.

Notably, all the isolates originating from the *L. vannamei* sample fell into Clusters A to C, except one into Cluster D. Similarly, the majority of the isolates (80.8 %) derived from *P. monodon* were grouped into Clusters A to D, with the remaining isolates belonging to Cluster H. Moreover, nine and two isolates from *P. monodon* exhibited 100 % similarity and fell into the same pulsotypes of Vpchn00033 and Vpchn00037, respectively. These results indicated more closely relationships of *V. parahaemolyticus* between *L. vannamei* and *P. monodon*, when compared to the other samples, in which the isolates belonging to seven of the eight PFGE clusters were identified (Fig. 5).

In addition, the isolates with MDR phenotypes that were derived from all the tested samples were distributed among the PFGE clusters. Based on the value of Simpson’s diversity index (0.9872), these isolates appeared to have the greater diversity in the *V. parahaemolyticus* population. As described above, the 90 isolates were resistant to Cr^3+^ and Zn^2+^, the majority of which also displayed resistance to Cu^2+^ (93.3 %), Pb^2+^ (87.8 %), and Cd^2+^ (73.3 %). The antibiotic and heavy-metal resistance phenotypes were widely distributed among the PFGE clusters with no significant relevance with the PFGE clusters, suggesting that resistance determinants perhaps spread among many genetic lineages within the *V. parahaemolyticus* population, regardless of different sample origins.

*V. parahaemolyticus* harbors two chromosomes (Makino et al. 2003). Mobile genetic elements carrying resistance genes have been identified from the bacterium (e.g., Song et al. 2013), which might be responsible for the large degree of variation in genotypes and resistance phenotypes among the isolates. It will be interesting to elucidate the precise mechanisms underlying the transmission of resistance determinants in *V. parahaemolyticus* population in the future research.

## Conclusions

In this study, a total of 400 *V. parahaemolyticus* isolates from commonly consumed fresh shrimps in Shanghai fish markets, China in 2013–2014 were isolated and characterized. Our data revealed an extremely low incidence of pathogenic *V. parahaemolyticus* carrying the two genes coding for the major virulence factors (*tdh* and *trh*, 0.0 and 0.5 %). However, high levels of antibiotic resistance were observed among the isolates against ampicillin (99 %), streptomycin (45.25 %), rifampicin (38.25 %), and spectinomycin (25.50 %). Moreover, approximately 15.3 % of the isolates exhibited MDR phenotypes. In addition, tolerance to heavy metals of Cr^3+^ and Zn^2+^ was observed in 90 antibiotic-resistant isolates, the majority of which also displayed resistance to Cu^2+^ (93.3 %), Pb^2+^ (87.8 %), and Cd^2+^ (73.3 %), when compared to *E. coli* K12. The PFGE-based genotyping of these isolates revealed a total of 71 pulsotypes, demonstrating remarkable genetic diversity of *V. parahaemolyticus* population in the shrimp samples, with the co-existence and wide distribution of a number of resistant isolates. The results also revealed the most contaminated reservoir of MDR *V. parahaemolyticus* in the *P. monodon* sample. The data in this study will refine our grasp of *V. parahaemolyticus* molecular ecology in aquaculture products and enable appropriate food-borne disease-control in aquaculture industry.
